# Diagnostic accuracy of the Alere Afinion AS100 point-of-care assay to screen for non-communicable diseases amongst people living with HIV

**DOI:** 10.1097/QAI.0000000000003098

**Published:** 2022-09-20

**Authors:** Nicola Bodley, Jienchi Dorward, Jessica Naidoo, Marothi Letsoalo, Kaminee Ramsaroop, Fathima Sayed, Yukteshwar Sookrajh, Paul K Drain, Nigel Garrett

**Affiliations:** 1 Centre for the AIDS Programme of Research in South Africa (CAPRISA), University of KwaZulu–Natal, Durban, South Africa; 2 Nuffield Department of Primary Care Health Sciences, University of Oxford, Oxford, UK; 3 eThekwini Municipality Health Unit, eThekwini Municipality, Durban, South Africa; 4 Department of Global Health, Schools of Medicine and Public Health, University of Washington, Seattle, USA; 5 Department of Medicine, School of Medicine, University of Washington, Seattle, USA; 6 Department of Epidemiology, School of Public Health, University of Washington, Seattle, USA; 7 Department of Public Health Medicine, School of Nursing and Public Health, University of KwaZulu-Natal, Durban, South Africa

**Keywords:** Afinion AS100, Diagnostic accuracy, people with HIV, non-communicable diseases, screening

## Introduction

Non-communicable diseases (NCDs), including hypertension, diabetes, and dyslipidaemias are risk factors for ischaemic heart disease and cerebrovascular accidents which are leading causes of death in low- and middle-income countries^1^. The prevalence of NCDs among the 7.8 million people living with HIV in South Africa is high^2^. Studies from a variety of settings have reported prevalences greater than 15% for hypertension, 3% for diabetes mellitus and 20% for dyslipidaemias^3–5^, highlighting the need for effective, integrated clinical services for both HIV and NCDs. In South Africa’s primary health care setting, results from specimens processed in central laboratories are often only available at the next clinic visit, which leads to multiple clinic visits and delays of 28 days or longer in counselling and management.

Effective solutions are needed to rapidly screen, diagnose and manage NCDs in antiretroviral therapy (ART) programmes. Point-of-care assays may allow quicker clinical decision making and accelerated entry into decentralised models of HIV and NCD care. Point-of-care HIV viral load testing has been shown to facilitate rapid identification of virological failure and improve outcomes. For NCDs, point-of-care lipid and glucose testing can provide an assessment of cardiovascular risk factors to allow clinicians to provide appropriate counselling and treatment for NCDs and HIV.

The Alere Afinion AS 100 Analyser (Abbott, Illinois, United States) is a small, electricity-powered, benchtop analyser for point-of-care testing of lipids and glycosylated haemoglobin (HbA1c), providing results from whole blood or plasma in less than 20 minutes. We aimed to evaluate the diagnostic accuracy of the Afinion analyser when used by nurses in a South African HIV clinic.

## Methods

We conducted a cross-sectional, prospective, clinic-based evaluation of the Afinion analyser. This was a sub-study of the STREAM (Simplifying HIV TREAtment and Monitoring) study^6,7^; a randomised controlled implementation trial of point-of-care viral load testing and task shifting in adults living with HIV and receiving ART at the Prince Cyril Zulu Communicable Disease Centre in central Durban, South Africa. The study was approved by the Biomedical Research Ethics Committee of the University of KwaZulu-Natal.

All STREAM participants who were identified as having a higher risk of cardiovascular disease according to South African guidelines,^8^ were invited to join the diagnostic accuracy sub-study. Risk factors included any of the following: Age >40 years, body mass index >30 kg/m^2^, previous diagnosis of hypertension, personal or strong family history of diabetes or cardiovascular disease, current smoker or receiving a protease inhibitor-based ART regimen. Testing was carried out at their study exit visit, 18 months after starting ART. The size of the cohort was based on resources and funds available for testing.

After obtaining informed consent, one of two research nurses collected a fingerprick capillary blood sample for point-of-care HbA1c and lipid panel testing using the Afinion analyser in the clinic. The Afinion analyser has a measurable range for each of the assays as follows, HbA1c 4.0-15.0%, total cholesterol 2.59-12.95 mmol/L, high density lipoprotein (HDL) 0.39-2.59 mmol/L and triglycerides 0.51-7.34 mmol/L.

The two clinical research nurses in the clinic underwent training by the manufacturer on the use of the point-of-care analyser. Corresponding venous blood samples were sent to a central laboratory for testing using the reference laboratory assays [DXC 600 clinical system by Beckman Coulter, Brea, California, United States for lipid measurements and the D10 Haemoglobin Analyser by Bio-Rad Laboratories, Hercules, California, United States for the HbA1c]. Both nurses provided feedback about the testing at the end of the study.

We compared sensitivity and specificity to detect abnormal results of the point-of-care assay with the reference laboratory analyser. Sensitivity and specificity were calculated using the exact binomial confidence limits using R software. We used Bland-Altman and Pearson correlation analyses to quantify the agreement between the two analysers. Correlations were carried out with Fisher transformation using the cor.test function in R. For the Bland-Altman results, no confidence intervals were reported for the bias estimates expect the limits of agreement. The resulting estimates (sensitivity, specificity, mean bias, and correlation) and their 95% confidence intervals were reported.

## Results

Of the 40 participants enrolled 50% were women, median age was 38 years (interquartile range [IQR] 31.5-45.0) and median body mass index was 29.2 kg/m^2^ (IQR 22.5-36.6). The mean CD4 count was 541 cells/uL (IQR 170 – 780 cells/uL). All participants were on firstline tenofovir based ART regimens, and none were receiving abacavir or a protease inhibitor. Paired samples for HbA1c were measured in all 40 participants, whereas only 38 participants had paired samples measured for total cholesterol, HDL and triglycerides, due to a shortage of Afinion cartridges available for those assays.

The prevalence of abnormal results using the reference laboratory assays was 15.0% for HbA1c ≥6.0 mmol/L, 28.9% for total cholesterol ≥5.0 mmol/L, 48.6% for HDL <2.0 mmol/L and 22.9% for triglycerides ≥1.7mmol/L. During analysis, there were three invalid triglyceride point-of-care results, which were outside the range measured by the Afinion analyser. HDL was not reported by the analyser for these results. Using the remaining valid results, the sensitivity and specificity of the Afinion to detect abnormal results were 83.3% and 97.7% for HbA1c (n=40), 72.7% and 96.3% for total cholesterol (n=38), 58.8% and 94.4% for HDL (n=35) and 100% and 70.4% for triglycerides (n=35) (Table 1).

Pearson correlation analysis of the point-of-care and laboratory assays for HbA1c was good (R=0.91) with a mean bias of -0.06. Correlation for total cholesterol was good (R=0.93) with a mean bias for the point-of-care analyser of -0.07. Correlation for HDL was good (R=0.91) with a mean bias of +0.17, while correlation for triglycerides was poor (R=0.51) with a mean bias of +0.89 (Table 1 and Supplementary Figures 1 and 2).

## Discussion

The Afinion analyser, is a multi-assay analyser capable of providing both HbA1c measurements and lipid profiles within minutes. Overall, the diagnostic accuracy of the Afinion assays were varied but this study placed the analyser in a true point-of-care setting where nurse led testing and interpretation was carried out. We identified a high prevalence of dyslipidaemia and abnormal HbA1c within this cohort with known cardiovascular risk factors, highlighting the need for targeted NCD screening at ART initiation and during monitoring visits for people living with HIV.

The Afinion analyser has previously been compared to other point-of-care and laboratory assays. A South African study, nested within the SHIOP (Sexual health, HIV infection and comorbidity with non-communicable diseases among Older Persons) study found that the Afinion analyser correlated well with the Abx Pentra 400 analyser (Horiba Medical, Montpellier, France) reference assay.[7] Their evaluation showed a sensitivity and specificity of 97.5% and 63.2% among males and 96.3% and 63.9% among females for HDL-cholesterol. It also found a sensitivity and specificity of 100% and 63.9% for total cholesterol, and 91.1% and 63.0% for triglycerides and 90.9% and 92.6% for HbA1c.^9^.

These findings, compared well with our study for HbA1c, but there were differences in the lipid results. One reason for this could be that their study used venous samples for both point-of-care and laboratory assays, whereas our study used capillary blood for point-of-care samples and venous blood for laboratory samples, because the Afinion has been marketed for use with fingerprick capillary samples, which are easier to take in primary care clinics than venous blood samples. Prior studies evaluating the lipid and lipoprotein concentrations of capillary versus venous blood samples have found that concentrations of lipids and lipoproteins were significantly lower in capillary versus venous blood serums.^10^

Another study, conducted in a community setting as part of a cardiovascular disease screening program in the United Kingdom, compared the Afinion analyser with the ADAMS A1C HA-8180V analyser (A. Menarini Diagnostics, Florence, Italy) for HbA1c and the Roche Modular P analyser (Roche Diagnostics, Mannheim, Germany) for lipids. That study also compared the Afinion to another previously validated point-of-care analyser, the Cholestech LDX (Abbott, Illinois, United States), and, like our study, used capillary samples for point-of-care analysis and venous samples for the reference assays. Overall, the study found that the Afinion analyser yielded results for lipid profiles in agreement with both the reference assay and the Cholestech LDX. The Afinion analyser also detected a similar number of new cases of diabetes mellitus based on HbA1c measurement compared to the reference assay^11^.

Limitations of our study included a small number of abnormal results to analyse and the discrepancy when comparing lipid contents between capillary blood for point-of-care assays against venous blood samples for the laboratory-based assays. However, overall, the assays performed sufficiently well in a clinic setting, and nurses in the study found the point-of-care analyser easy to use, which indicates that this assay may be an acceptable tool for the primary healthcare setting in low- and middle-income countries.

While more NCD screening is necessary, additional research is needed on how best to integrate point-of-care technology into existing HIV and primary healthcare programmes. The use of point-of-care tests in HIV/ART clinics could help to identify more patients who need NCD care. Furthermore, it would likely reduce the number of required clinic visits, because management could be initiated on the same day as testing. This could be more convenient to patients, thereby potentially improving adherence and long-term treatment outcomes for HIV and NCDs.

In conclusion, the Afinion analyser shows promise as a point-of-care analyser for NCDs, with varied strength of correlations in our study. Larger studies may be needed to further assess the accuracy and cost-effectiveness of this assay in a programmatic setting.

## Supplementary Material

Supplementary Figures 1 and 2

## Figures and Tables

**Table 1:** Accuracy of the Alere Afinion AS100 point-of-care assays compared against reference assays

Assay	N, LAB (POC)	Reference (Abnormal)	Sensitivity (95% CI)	Specificity (95% CI)	Mean bias (95% CI of LoA)	Correlation R (95% CI)
Haemoglobin A1c	40 (40)	>=6.0%	83.3 (35.9,99.6)	97.1 (84.7,99.9)	-0.06 (-0.48,0.36)	0.91 (0.83,0.95)
Total cholesterol	38 (38)	>=5.0 mmol/L	72.7 (39.0,94.0)	96.3 (81.0,99.9)	-0.07 (-0.86,0.72)	0.93 (0.87,0.96)
HDL cholesterol	38 (35)	<1.2 mmol/L	58.8 (32.9,81.6)	94.4 (72.7,99.9)	0.17 (-0.16,0.49)	0.91 (0.83,0.96)
Triglycerides	38 (35)	>=1.7mmol/L	100 (63.1,100)	70.4 (49.8,86.2)	0.89 (-1.58,3.35)	0.51 (0.21,0.72)
