# Supplementary material for: Diagnostic accuracy of the Alere Afinion AS100 point-of-care assay to screen for non-communicable diseases amongst people living with HIV
Source: J Acquir Immune Defic Syndr. Author manuscript; Available in PMC 2023 Jan 6. (PMC9742175; doi:10.1097/QAI.0000000000003098)
Supplement: Supplementary Figures 1 and 2 [file EMS157958-supplement-Supplementary_Figures_1_and_2.docx]

**Supplementary Figure 1: Mean bias and limits of agreement comparing** **the Alere Afinion AS100** **point-of-care assay (POC) with reference laboratory assays (LAB)**


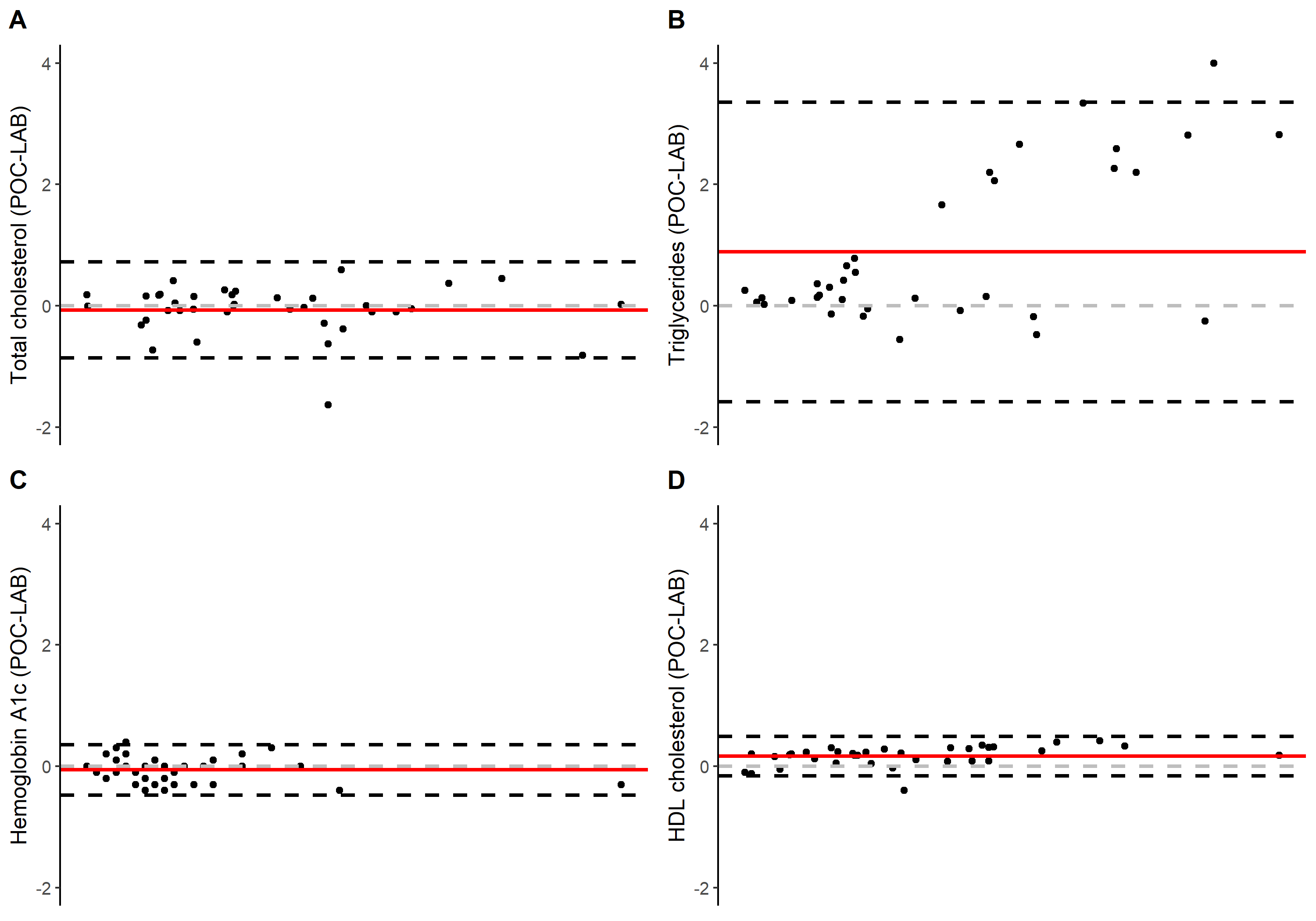


***Figure 1:*** The dashed gray line is the zero line that represents a zero bias in a case of a perfect match between point-of-care assay (POC) and reference laboratory assays (LAB) results. The closer the bias line (red solid line) is to the dashed line, the less the bias or more agreement there is between the two assays. The black dashed lines are the lower and upper limits of agreement between the two assays. The dots represent the differences between the POC and LAB results.

**Supplementary Figure 2: Correlation of the Alere Afinion AS100 point-of-care assay (POC) with reference laboratory assays (LAB)**


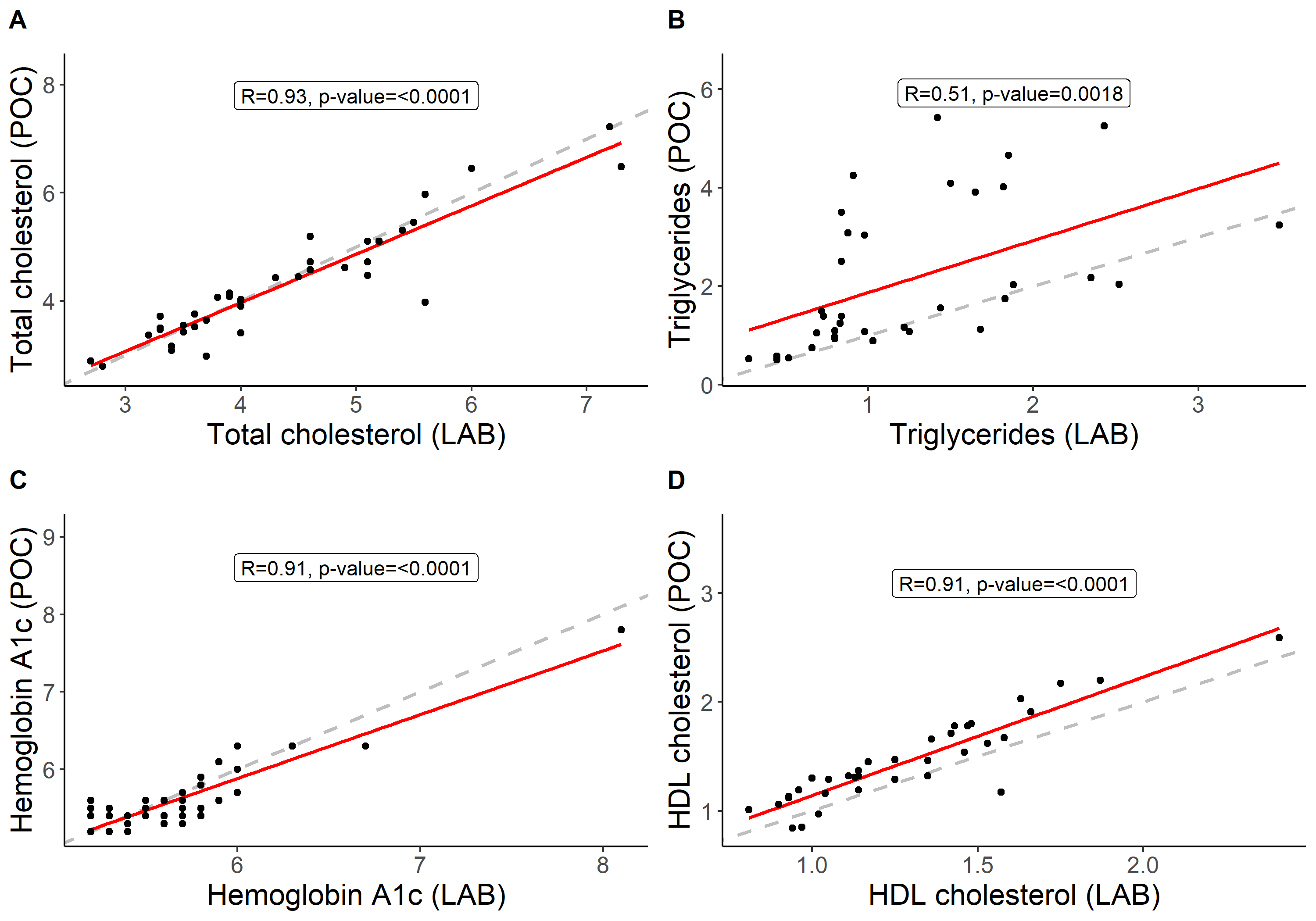


**Figure 2**: The dashed grey line is the 45-degrees line that represents the perfect correlation. The closer the fitted line (red solid line) is to the dashed line, the higher the correlation. The dots represent the observed point-of-care assay (POC) and reference laboratory assays (LAB) results.
